# Super elderly patients with intertrochanteric fractures do not predict worse outcomes and higher mortality than elderly patients: a propensity score matched analysis

**DOI:** 10.18632/aging.103466

**Published:** 2020-07-10

**Authors:** Junfei Guo, Zhiqian Wang, Mingming Fu, Jun Di, Junpu Zha, Junchuan Liu, Guolei Zhang, Qingxian Wang, Hua Chen, Peifu Tang, Zhiyong Hou, Yingze Zhang

**Affiliations:** 1 Department of Orthopaedic Surgery, Third Hospital of Hebei Medical University, Shijiazhuang 050051, Hebei, P.R. China; 2 Department of Orthopaedic Surgery, Chinese PLA General Hospital, 301 Hospital, Beijing 100853, P.R. China; 3 Chinese Academy of Engineering, Beijing 100088, P.R. China

**Keywords:** super elderly, intertrochanteric, mortality, functional outcomes, propensity score matched

## Abstract

We aimed to investigate whether super elderly patients aged over 90 years had significantly worse functional outcomes, perioperative complications, and survival rates. Among 3560 patients aged over 65 years presenting with intertrochanteric fractures and treated surgically between Jan 2014 and Jan 2019, 2242 patients were included, including 206 in super elderly group and 2036 in elderly group. After using propensity score matching to minimize the effects of possible confounding variables, 192 remained in each group. No significant difference was observed in functional outcomes, perioperative complications, or 30-day, 90-day, and 1-year mortality after propensity score matching and McNemar’s tests (*p*>0.05). After an average follow-up of 37 months, the Kaplan-Meier survival curve showed no significant difference between the two groups in terms of cumulative survival rate (*p*=0.081, log-rank). Our data demonstrated progressive increases in mortality and poor outcomes with increasing Elixhauser comorbidity scores, which represented the severity index of patients preoperatively. Our study also found that there were weak correlations between five characteristics and the patient age. These results all suggested that it is not the advanced age itself but other concomitant factors, that appear to be responsible for the adverse functional outcomes, perioperative complications, and mortality in super elderly patients.

## INTRODUCTION

With the aging of the population, the prevalence and incidence of hip fractures have increased rapidly, particularly in developing countries. It has been reported that the number of elderly Chinese people aged over 60 had reached 249 million, accounting for 17.9% of the total population by 2018, and it has been estimated that by 2050, that number will exceed 450 million, comprising over 30% of the total population [1]. Wang et al. [2] also reported that the elderly population in China comprised nearly 20% of the elderly population worldwide. Intertrochanteric fractures, as one of the major orthopedic clinical problems in China that is a common cause of illness, disability and mortality in the elderly people, leads to a heavy socioeconomic pressure on society.

According to the literature, the postoperative recovery for these fragile patients with intertrochanteric fractures is not to be well expected due to preexisting severe comorbidities, delay of surgery, anemia and adverse reactions following anesthesia and blood transfusion, which are associated with a high risk of perioperative complications and mortality and thus can be a catastrophic event precipitating a steep decline in health and independence [3–10]. To the best of our knowledge, the possible role of age is still under discussion. Several previous publications [11–15] have shown that functional impairment increases with age, while others [16–20] have suggested that age is not associated with adverse outcomes. However, relevant research on whether super elderly patients aged over 90 years have significantly worse functional outcomes and survival rates than others is still limited.

The objective of this study was to evaluate the population of super elderly patients aged over 90 years with intertrochanteric fractures and treated with intramedullary fixation on the functional outcomes, perioperative complications, and mortality.

## RESULTS

**Figure 1:**
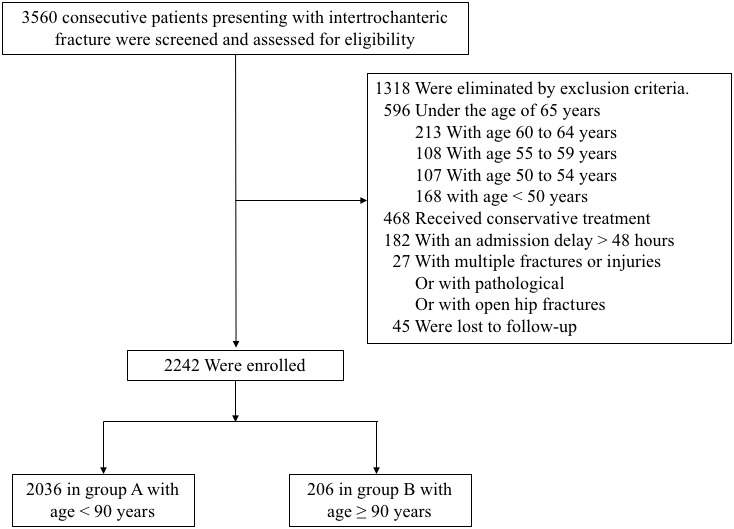
**Flow diagram of included patients.**

From Jan 2014 to Jan 2019, a total of 3560 consecutive patients presenting with intertrochanteric fracture were screened and assessed for eligibility to participate in this study. A total of 1318 patients were eliminated by the exclusion criteria. Among these, 596 patients were under the age of 65 years (213 with age 60 to 64, 108 with age 55 to 59, 107 with age 50 to 54, 168 with age < 50); 468 patients (46 with age ≥ 90, 422 with age 65-89) received conservative treatment; 182 patients (22 with age ≥ 90, 160 with age 65-89) had an admission delay of more than 48 hours; 27 patients had multiple fractures or injuries, pathological or open hip fractures; and 45 patients were lost to follow-up. Finally, 2242 patients, including 2036 in group A with age < 90 years and 206 in group B with age ≥ 90 years, presenting with intertrochanteric fracture met our inclusion and exclusion criteria (Figure 1).

The baseline characteristics of patients in the two groups are summarized in Table 1. More than half of the fractures were stable and the majority of the patients were female in both groups. Characteristics including the American Society of Anesthesiologists (ASA) grade, body mass index (BMI), Hb level at admission, type of anesthesia, volume of intraoperative blood loss, transfusion requirement, and Elixhauser comorbidity scores [21, 22] were significantly different between the two groups. After propensity score weighting, there were 192 matched patients in each group, and the baseline characteristics were balanced between the two cohorts in terms of all covariates (Table 1).

**Table 1:** Patient characteristics at baseline.^§^

**Variables**	**Pre-matching**			**Post-matching**		
	**Group A (< 90, n=2036)**	**Group B (≥ 90, n=206)**	***p* value**	**Group A (< 90, n=192)**	**Group B (≥ 90, n=192)**	***p* value**
Gender (female)	1364 (67.0%)	150 (72.8%)	0.089	138 (71.9%)	140 (72.9%)	0.819
Fracture type (stable)	1112 (54.6%)	105 (51.0%)	0.317	96 (50.0%)	98 (51.0%)	0.838
Fracture side (left)	1083 (53.2%)	98 (47.6%)	0.124	87 (45.3%)	95 (49.5%)	0.414
ASA grade (1/2/3/4/5)	**400/600/724/269/43**	**31/53/80/31/11**	**0.017**	37/48/74/25/8	31/52/75/25/9	0.944
BMI (normal/overweight/obesity)	**1304/569/163**	**151/49/6**	**0.006**	145/41/6	141/45/6	0.886
Anesthesia (general)	**782 (38.4%)**	**61 (29.6%)**	**0.013**	68 (35.4%)	58 (30.2%)	0.277
Red blood cell transfusion	**1519 (74.6 %)**	**184 (89.3%)**	**<0.001**	171 (89.1%)	170 (88.5%)	0.871
Hb level at admission (>12/10-12/8-10/<8)	**616/862/460/98**	**28/73/85/20**	**<0.001**	30/68/74/20	28/71/80/13	0.604
Elixhauser comorbidity scores (<0/0/1-5/6-13/≥14)	**38/1040/319/560/79**	**7/75/53/51/20**	**<0.001**	5/84/33/58/12	7/74/52/45/14	0.135
Surgery delay	6.0 ± 3.2	6.0 ± 3.1	0.847	6.0 ± 3.0	6.0 ± 3.1	0.921
Duration of operation	99.4 ± 35.5	95.9 ± 31.5	0.123	90.6 ± 29.7	95.3 ±31.9	0.128
Intraoperative blood loss	**200 (100, 300)**	**200 (100, 262.5)**	**0.001**	200 (100, 300)	200 (100, 300)	0.955

^§^Values are presented as the number (%). Surgery delay and duration of operation are presented as the mean ± standard deviation. Intra-operative blood loss is presented as the median (interquartile range). **Bold** means that values are statistically significant.

Pre-matching and post-matching results, including perioperative complications, functional outcomes, and 30-day, 90-day, and 1-year mortality, are shown in Table 2. The statistical distribution showed that perioperative complications, including severe complications, cardiac complications, pulmonary complications, endocrine/metabolic complications and outcomes, were not significantly different between the two groups after propensity score matching (PSM), although the differences were significant before PSM (*p*< 0.001, *p*< 0.001, *p*< 0.001, *p* = 0.026 and *p*< 0.001, respectively). No significant difference was observed in other complications, such as neurological complications and hematological complications, before and after PSM. The crude mortality rate was 0.89% at 30 days, with no significant difference between the two groups before and after PSM. The mortality rates at 90 days and 1 year were higher in patients aged ≥ 90 years than in patients aged < 90 years group (*p*=0.015 and *p*<0.001). However, there was no significant difference between the two groups after PSM.

**Table 2:** Patient complications and outcomes.^§^

**Variables**	**Pre-matching**				**Post-matching**			
	**Group A (< 90, n=2036)**	**Group B (≥ 90, n=206)**	***p* value**	**Group A (< 90, n=192)**	**Group B (≥ 90, n=192)**	***p* value**	**OR**	**95% CI**
Severe complications	**431 (16.6%)**	**54 (35.0%)**	**<0.001**	40 (20.8%)	47 (24.5%)	0.393	1.232	0.763 to 1.989
Cardiac complications	**431 (21.2%)**	**72 (35.0%)**	**<0.001**	58 (30.2%)	65 (33.9%)	0.444	1.182	0.770 to 1.816
Pulmonary complications	**181 (8.9%)**	**47 (22.8%)**	**<0.001**	27 (14.1%)	37 (19.3%)	0.171	1.459	0.848 to 2.509
Neurological complications	114 (5.6%)	18 (8.7%)	0.068	14 (7.3%)	17 (8.9%)	0.574	1.235	0.591 to 2.582
Hematological complications	874 (42.9%)	94 (45.6%)	0.455	86 (44.8%)	83 (43.2%)	0.758	0.939	0.627 to 1.404
Endocrine/metabolic complications	**1399 (68.7%)**	**157 (76.2%)**	**0.026**	143 (74.5%)	143 (74.5%)	1.000	1.000	0.632 to 1.582
30-day mortality	17 (0.8%)	3 (1.5%)	0.607	3 (1.6%)	2 (1.0%)	0.653	0.663	0.110 to 4.014
90-day mortality	**28 (1.4%)**	**8 (3.9%)**	**0.015**	4 (2.1%)	7 (3.6%)	0.359	1.778	0.512 to 6.177
1-year mortality	**125 (6.1%)**	**28 (13.6%)**	**<0.001**	15 (7.8%)	23 (12.0%)	0.172	1.606	0.811 to 3.182
Functional Outcomes								
independent walking	**842 (41.4%)**	**56 (27.2%)**	**<0.001**	71 (37.0%)	55 (27.1%)	0.082	0.684	0.446 to 1.050
use of walking aids	629 (30.9%)	66 (32.0%)	0.735	57 (29.7%)	59 (32.3%)	0.824	1.051	0.680 to 1.624
wheelchair	116 (5.7%)	16 (7.8%)	0.229	13 (6.8%)	15 (7.8%)	0.695	1.167	0.540 to 2.523
bedridden	54 (2.7%)	6 (2.9%)	0.825	4 (2.1%)	6 (3.1 %)	0.522	1.516	0.421 to 5.460
death	**395 (19.4%)**	**62 (30.1%)**	**<0.001**	47 (24.5%)	57 (29.7%)	0.251	1.303	0.829 to 2.046

^§^Values are presented as the number (%). **Bold** means that values are statistically significant.

For relationships between the Elixhauser comorbidity score and survival state/functional outcomes, the data shown in Table 3 demonstrated progressive increases in mortality and poor functional outcomes with increasing Elixhauser comorbidity score, which represented the severity index of patients preoperatively (*p*<0.05). By using the Spearman method, Table 4 showed the weak correlation between patient age and the factors of ASA grade, Elixhauser comorbidity score, Hb level at admission, intraoperative blood loss, and transfusion requirement.

**Table 3:** The association of Elixhauser comorbidity scores with functional outcomes and mortality.^§^

**Variables**	**Elixhauser comorbidity scores**					
	**<0 (n=45)**	**0 (n=1115)**	**1-5 (n=372)**	**6-13 (n=611)**	**≥14 (n=99)**	***p* value**
survival state (dead)						
30 days	**0 (0.0%)**	**3 (0.3%)**	**3 (0.8%)**	**11 (1.8%)**	**3 (3.0%)**	**0.003**
90 days	**0 (0.0%)**	**11 (1.0%)**	**7 (1.9%)**	**13 (2.1%)**	**5 (5.1%)**	**0.028**
1 year	**1 (2.2%)**	**57 (5.1%)**	**36 (9.7%)**	**47 (7.7%)**	**11 (11.1%)**	**0.005**
functional outcomes						**0.017**
independent walking	**25 (55.6%)**	**476 (42.7%)**	**129 (34.7%)**	**235 (38.5%)**	**33 (33.3%)**	
use of walking aids	**8 (17.8 %)**	**333 (29.9%)**	**111 (29.8%)**	**210 (34.4%)**	**33 (33.3%)**	
wheelchair	**1 (2.2%)**	**64 (5.7%)**	**28 (7.5%)**	**30 (4.9%)**	**9 (9.1%)**	
bedridden	**3 (6.7%)**	**23 (2.1%)**	**11 (3.0%)**	**19 (3.1%)**	**4 (4.0%)**	
death	**8 (17.8%)**	**219 (19.6%)**	**93 (25.0%)**	**117 (19.1%)**	**20 (20.2%)**	

^§^Values are presented as the number (%). **Bold** means that values are statistically significant.

**Table 4:** The association of age group with ASA grade, Elixhauser comorbidity score, Hb level at admission, intraoperative blood loss, transfusion requirement, and anesthesia type.^§^

**Variables**	**Age group**					
	**65 – 69 (n=236)**	**70 – 79 (n=874)**	**80 – 89 (n=926)**	**90 – 99 (n=198)**	**≥ 100 (n=8)**	**Spearman’s r Statistic**	***p* value**
ASA grade						**0.052**	**0.015**
1	**47 (10.9%)**	**182 (42.2%)**	**171 (39.7%)**	**29 (6.7%)**	**2 (0.5%)**		
2	**73 (11.2%)**	**255 (39.1%)**	**272 (41.7%)**	**51 (7.8%)**	**2 (0.3%)**		
3	**74 (9.2%)**	**318 (39.6%)**	**332 (41.3%)**	**78 (9.7%)**	**2 (0.2%)**		
4	**38 (12.7%)**	**103 (34.3%)**	**128 (42.7%)**	**29 (9.7%)**	**2 (0.7%)**		
5	**4 (7.4%)**	**16 (29.6%)**	**23 (42.6%)**	**11 (20.4%)**	**0 (0.0%)**		
Elixhauser comorbidity score						**0.071**	**0.001**
<0	**9 (20.0%)**	**10 (22.2%)**	**19 (42.2%)**	**6 (13.3%)**	**1 (2.2%)**		
0	**127 (11.4%)**	**471 (42.2%)**	**442 (39.6%)**	**72 (6.5%)**	**3 (0.3%)**		
1-5	**20 (5.4%)**	**133 (35.8%)**	**166 (44.6%)**	**52 (14.0%)**	**1 (0.3%)**		
6-13	**74 (12.1%)**	**236 (38.6%)**	**250 (40.9%)**	**48 (7.9%)**	**3 (0.5%)**		
≥14	**6 (6.1%)**	**24 (24.2%)**	**49 (49.5%)**	**20 (20.2%)**	**0 (0.0%)**		
Hb level at admission						**0.237**	**<0.001**
>12	**103 (16.0%)**	**302 (46.9%)**	**211 (32.8%)**	**27 (4.2%)**	**1 (0.2%)**		
10-12	**92 (9.8%)**	**384 (41.1%)**	**386 (41.3%)**	**68 (7.3%)**	**5 (0.5%)**		
8-10	**34 (6.2%)**	**156 (28.6%)**	**270 (49.5%)**	**83 (15.2%)**	**2 (0.4%)**		
<8	**7 (5.9%)**	**32 (27.1%)**	**59 (50.0%)**	**20 (16.9%)**	**0 (0.0%)**		
Intraoperative blood loss	**200 (100, 300)**	**200 (100, 300)**	**200 (100, 300)**	**200 (100, 300)**	**150 (57.5, 200)**	**-0.088**	**<0.001**
Red blood cell transfusion						**0.203**	**<0.001**
No	**105 (44.5%)**	**245 (28.0%)**	**167 (18.0%)**	**21 (10.6%)**	**1 (12.5%)**		
Yes	**131 (55.5%)**	**629 (72.0%)**	**759 (82.0%)**	**177 (89.4%)**	**7 (87.5%)**		
Anesthesia						0.019	0.370
General	95 (11.3%)	322 (38.2%)	365 (43.3%)	58 (6.9%)	3 (0.4%)		
Regional	141 (10.1%)	552 (39.5%)	561 (40.1%)	140 (10.0%)	5 (0.4%)		

^§^Values are presented as the number (%). Intra-operative blood loss is presented as the median (interquartile range). **Bold** means that values are statistically significant.

**Figure 2:**
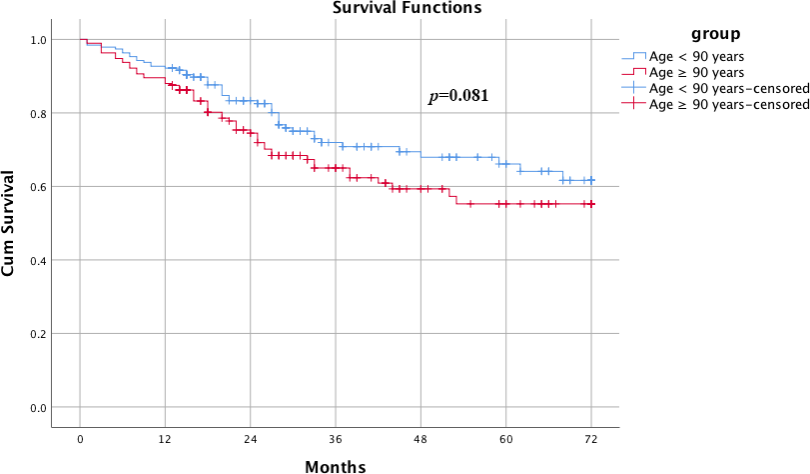
**Kaplan-Meier survival curves for elderly and super elderly patients after hip surgery.** Patients older than 90 years had a risk of death at 1 year that was 0.98 time as high, a risk of death at 2 years that was 1.6 times as high, a risk of death at 3 years that was 1.5 times as high and a risk of death at 4 years that was 1.3 times as high as the risk compared with the patients less than 90 years. However, the Kaplan-Meier survival curve showed no significant difference between the two groups of patients on cumulative survival rate. (*p*=0.081, log-rank).

The average follow-up was 37 months. The overall mortality rate of all patients was 20.38% at the end of this study, and deaths occurred predominantly during the first 4 years. The cumulative survival rates of the two groups of patients were gradually decreased within the first three years after injury and stabilized thereafter. Patients older than 90 years had a risk of death at 1 year that was 0.98 times as high, a risk of death at 2 years that was 1.6 times as high, a risk of death at 3 years that was 1.5 times as high and a risk of death at 4 years that was 1.3 times as high as the risk of the patients less than 90 years. However, the Kaplan-Meier survival curve showed no significant difference between the two groups of patients in cumulative survival rate. (Figure 2, *p*=0.081, log-rank).

## DISCUSSION

In the current study involving 2242 patients, we observed that in general, there was a higher ASA grade, Elixhauser comorbidity scores, and transfusion rates and a lower Hb level at admission, BMI, volume of intraoperative blood loss and percentage of general anesthesia in super elderly patients (with age ≥ 90 years) than in younger patients (with age < 90 years). However, after PSM and McNemar’s tests, we confirmed that super elderly patients do not predict a worse clinical outcomes in terms of functional outcomes, perioperative complications, and mortality than younger patients. Additionally, the discrepancy of all outcomes can likely be attributed to the differences in the baseline characteristics of the study populations. In other words, age is not a well-studied prognostic factor for patients’ functional outcomes, perioperative complications, and mortality, especially in super elderly patients based on the results of this study.

Functional outcomes after hip fracture surgeries are likely to be multifactorial. Numerous studies [11–14, 16] have shown that age, gender, ASA-grade, transfusion requirement/volume and comorbidities were predictors of morbidity and mortality after surgery for hip fractures. In addition, the impact of other factors, such as surgery delay, type of anesthesia, duration of operation, and volume of intraoperative blood loss, have not been consistently demonstrated. For example, some studies [13, 16, 23, 24] suggested better survival states and lower morbidity rates with early surgery while other studies [25, 26] suggested no difference due to surgery delay. Another example is that the choice of anesthesia for hip fracture surgery in elderly patients is still controversial [10, 27–30]. However, it should be noted that while the age factor has been shown to be associated with mortality after hip fracture surgery, it is not always adjusted for other covariables that may confound survival analyses.

In this study, we used PSM to minimize the effects of possible confounding variables. The differences represented the effect of age on ASA grade, BMI, Hb level at admission, type of anesthesia, volume of intraoperative blood loss, transfusion requirement, and Elixhauser comorbidity scores before PSM were eliminated in the present study. Our result implies that it is not advanced age itself but other factors associated with age that appear to be responsible for adverse outcomes and mortality in elderly patients, which is consistent with Sophia et al. [17]. In a review of twelve studies based on large databases that addressed the influence of age on outcome, Sophia et al. [17] revealed that it is not age itself but concomitant factors, such as severity of illness and premorbid functional status, that might influence prognosis. Similarly, several previous studies [18–20] using multivariate analyses also concluded that age was not an independent risk factor for mortality. A cohort study performed by Somme et al. [20] also found that advanced age is not directly associated with mortality. Compared to these former studies, the strength of our study lies in the more recent data included from 2014 to 2019, with a relatively large sample size.

According to the baseline characteristics of patients in the two groups, a greater percentage of patients had an ASA grade 4 or 5 and an Elixhauser comorbidity score of 6 or higher in the super elderly patient group than in the elderly patient group, indicating that the older the patients, the more severe the comorbidities they had. In view of this, early identification of super elderly individuals prone to postoperative morbidity and mortality and accelerated care, consisting of rapid medical optimization, are of vital importance. Previous studies have shown that good postoperative recovery for elderly patients with hip fractures was not expected because of poor surgical tolerance and tendency for anemia, which were associated with significant adverse clinical outcomes [3, 4, 31, 32]. Gruson et al. [31] found that 45.6% of emergency surgical patients had anemia, and unexplained anemia accounted for approximately one-third of these patients [3]. In the current study, there were significantly more patients with an Hb level of 10 g/dL or less at admission in the super elderly group than in the elderly group (51.0% vs. 27.4%). Among surgery-related factors, patients aged 90 years or older received more regional anesthesia and had more transfusion requirements but less intraoperative blood loss than the younger patients. After using the Spearman method, we further demonstrated that the aforementioned factors of ASA grade, Elixhauser comorbidity score, Hb level at admission, intraoperative blood loss, and transfusion requirement, even though there was only a weak correlation, were related to the patient age. However, we did not find any significant relationship between anesthesia type and age, despite regional anesthesia being most common in the 90 years or older age group.

Although many published articles [6, 7, 33, 34] have previously performed survival analyses and studied the short-term outcomes of elderly patients with hip fractures, longer-term survival and functional outcomes are less frequently reported. In our seven-year follow-up, 457 of 2242 total patients died at the end of the study, and the mortality rates of super elderly patients and elderly patients were 30.1% and 19.4% before PSM, respectively. Our data suggest that the mortality rate is lower than previous findings [6, 7, 33]. This can be ascribed to the patients who receive conservative treatment being excluded due to the severe comorbidities, and we restricted the study population to surgical patients. After PSM, the survival analysis showed that there was no significant difference between the two groups of patients in terms of cumulative survival rate. (*p*=0.081, log-rank). Marginally significant results (*p*-value close to 0.05) should be interpreted with caution; however, there was also a marginally significant trend towards a difference in survival rate between the two groups. There is possible selection bias, and caution should therefore be taken when generalizing related results and conclusions.

Surprisingly, we found that most patients could walk independently or with the help of walking aids, while only a relatively small percentage of patients were restricted to wheelchairs or beds. Ekstrom et al. [34] concluded that only approximately 55% of patients regain their previous walking ability, and approximately 34% of patients lose their previous level of daily living function. Based on our data, 27.2% of super elderly patients and 41.4% of elderly patients walked independently before PSM, while the percentages were 27.1% and 37.0%, respectively, after PSM. In addition, a notable proportion of individuals are able to walk independently but still require help with walking aids to avoid falling again, which leads to a decrease in the independent walking status ratio.

Many previous studies have assessed the factors that influence the functional outcomes and mortality rates of elderly hip fracture patients. However, this is the first study to assess the effect of age on functional outcomes, perioperative complications, and mortality in super elderly patients with intertrochanteric hip fracture. Such quantitative analyses might increase the orthopedist’s confidence in treatment management for super elderly patients and be beneficial for clinicians looking to establish probabilities for adverse results in the future and making and establishing rational goals of medical care for this patient population. The strength of this study includes an appropriate comprehensive preoperative evaluation of functional status using Elixhauser comorbidity scores, a validated and simple scoring system that accounts for 30 comorbidities. Other strengths are the PSM method we used and the specific cohort of patients who received surgery by single internal fixation, which eliminated the effects of possible confounding variables. Finally, the cohort comprises a relatively large number of patients and has a long-term follow-up. The limitations of this study include its retrospective design and the data being collected in a single center. In addition, we did not control all risk factors, such as smoking and other unknown confounders, including perioperative laboratory values and surgeon practice, for analysis, which may potentially influence the findings. However, this is the first study including multiple relative contributions of patient, fracture, surgical, anesthetic and transfusion factors, which have not been studied together.

In conclusion, super elderly patients aged more than 90 years do not predict worse clinical outcomes in terms of functional outcomes, perioperative complications, and mortality than elderly patients aged 65-90 years treated surgically with proximal femoral nail anti-rotation (PFNA) for intertrochanteric fractures.

## MATERIALS AND METHODS

### Patients and groups

The study population consisted of all patients aged 65 years or older presenting with intertrochanteric fractures at a single Level I trauma center in China between Jan. 2014 and Jan. 2019. We included patients who were 65 years or older, injured by falling, had an admission delay of injury less than 48 hours, underwent hip surgery of closed reduction and internal fixation by PFNA, and received a minimum of one-year follow-up. Patients who had multiple fractures or injuries or pathological or open hip fractures, received conservative treatment due to severe comorbidities or refused surgery were excluded. Patients were retrospectively assigned to two groups according to their age: group A with patients < 90 years old and group B with patients ≥ 90 years old. This study was approved by the institutional internal review board of the participating institution.

The following patient characteristics were extracted: gender, fracture type (stable or unstable according to the AO/OTA classification), fracture side (left or right), body mass index (BMI, normal with BMI<24 kg/m^2^, overweight with 24≤BMI<28 kg/m^2^ and obesity with BMI≥28 kg/m^2^), American Society of Anesthesiologists grade (ASA, six grade), surgery delay (defined as the time between an injury to definitive surgery), hemoglobin (Hb) level at admission, type of anesthesia (general anesthesia or regional anesthesia), duration of operation, volume of intraoperative blood loss, and transfusion requirement. The Elixhauser comorbidity method was used in this study cohort to identify the patients’ comorbidities at admission by the sum of weighted points based on 30 different medical conditions (including congestive heart failure, cardiac arrhythmias, valvular disease, pulmonary circulation disorders, peripheral vascular disorders, hypertension, paralysis, neurodegenerative disorders, chronic pulmonary disease, uncomplicated diabetes, complicated diabetes, hypothyroidism, renal failure, liver disease, peptic ulcer disease, AIDS/HIV, lymphoma, metastatic cancer, solid tumor without metastasis, rheumatoid arthritis/collagen vascular disease, coagulopathy, obesity, weight loss, fluid and electrolyte disorders, blood loss anemia, deficiency anemia, alcohol abuse, drug abuse, psychosis and depression).

### Outcome assessments

Each patient was followed up from enrollment in the cohort to the date of death or the end of the study, whichever was earlier. Time and the leading cause of death were recorded. Then, 30-day, 90-day, and 1-year mortality and functional outcomes, including independent walking, use of walking aids, wheelchair, bedridden status, and death, were also recorded. Perioperative complications classified as severe complications (consisting of pulmonary embolisms, sudden cardiac death, stroke, acute myocardial infarction, acute cerebral infarction, heart failure, respiratory failure, and stress ulceration), cardiac complications (consisting of new-onset cardiac arrhythmia, ischemic heart disease, hemodynamic instability, low cardiac output, and cardiac dysfunction), pulmonary complications (consisting of pulmonary infection, respiratory insufficiency, pleural effusion, and atelectasis), neurological complications (consisting of transient ischemic attack and delirium), hematological complications (consisting of deep vein thrombosis and anemia) and endocrine/metabolic complications (consisting of stress hyperglycemia, electrolyte disorder, hypoproteinemia, and metabolic or endocrine disturbances) were also recorded.

### Statistical analysis

The continuous variables were evaluated for normality by using the Shapiro-Wilk test. Data satisfying normality are presented as the mean ± standard deviation; otherwise, they are presented as the median (interquartile range). Categorical data are presented as absolute numbers (percentages), and the chi-square test was used to compare patient number distributions between the groups. The tests for significant differences between normally distributed data samples were performed using Student’s t test, while nonnormally distributed groups were compared using the Mann-Whitney U test. To minimize selection bias and potential confounding effects, we carried out adjustments for differences in baseline characteristics between the two groups by means of propensity score matching (PSM) using a 1:1 ratio. PSM was performed via the caliper matching method, and the propensity score was calculated by logistic regression analysis using covariates including gender, fracture type, fracture side, BMI, ASA grade, surgery delay, Hb level at admission, type of anesthesia, duration of operation, volume of intraoperative blood loss, transfusion requirement and Elixhauser comorbidity scores. After PSM, McNemar’s tests were used to examine the association of super elderly patients with outcomes, perioperative complications, and 30-day, 90-day, and 1-year mortality after hip surgery. Finally, the Spearman correlation of patient age and the factors of ASA grade, Elixhauser comorbidity score, Hb level at admission, intraoperative blood loss, transfusion requirement, and anesthesia type which may be influenced by age, were computed. All data analyses were performed using IBM SPSS Statistics for Windows, version 26.0 (IBM, Armonk, NY, USA). The level of significance was set at *p*< 0.05.
